# Gender Affirming Revision Vaginoplasty Utilizing Decellularized Fish Skin Xenograft: Surgical Technique and Outcomes

**DOI:** 10.1590/S1677-5538.IBJU.2025.0340

**Published:** 2025-08-30

**Authors:** Seyed Sajjad Tabei, Alex J. Xu, Rachel Pope, Kyle Scarberry, Kirtishri Mishra, Shubham Gupta

**Affiliations:** 1 University Hospitals Cleveland Medical Center Urology Institute Cleveland Ohio United States Urology Institute, University Hospitals Cleveland Medical Center, Cleveland, Ohio, United States; 2 Case Western Reserve University School of Medicine Department of Urology Cleveland Ohio United States Department of Urology, Case Western Reserve University School of Medicine, Cleveland, Ohio, United States; 3 The MetroHealth System Division of Urology Cleveland Ohio United States Division of Urology, The MetroHealth System, Cleveland, Ohio, United States

**Keywords:** Gender-Affirming Surgery, Genitourinary Agents, Robotic Surgical Procedures

## Abstract

**Purpose:**

Tissue availability for revision vaginoplasty in trans feminine individuals is often limited, creating challenges in cases with prior surgery or inadequate local tissue. While not yet extensively studied in genital reconstruction, Kerecis SurgiClose^®^, a decellularized fish skin xenograft, has shown promise as a skin substitute in other surgical contexts, including burn care and chronic wound management. This study aims to describe the surgical technique and evaluate the short-term clinical outcomes of revision vaginoplasty using decellularized fish skin xenograft (FSXRV) in trans feminine individuals.

**Materials and Methods:**

A retrospective review of 27 trans feminine patients who underwent FSXRV between February 2023 and December 2024 was conducted. Data on preoperative characteristics, intraoperative details, and postoperative outcomes were collected from electronic medical records and analyzed.

**Results:**

The median age was 36 years, and the median BMI was 27 kg/m^2^. Median postoperative follow-up was 261 days. Indications for FSXRV included loss of neovaginal depth (81.5%), proximal introital narrowing (14.8%), and devitalized neovaginal grafts (3.7%). The xenograft was applied in various configurations and anatomical locations using either a perineal-only or combined perineal-robotic approach. No intraoperative or major (Clavien-Dindo ≥II) complications occurred within 30 days postoperatively. Five patients (18.5%) underwent subsequent canal revision after FSXRV. Positive outcomes were reported in 74% of patients using the Patient Global Impression of Improvement scale.

**Conclusion:**

Fish skin xenograft revision vaginoplasty demonstrated early safety and feasibility for revision neovaginal lining and appeared to reduce reliance on autologous grafts or flaps in complex revision settings.

## INTRODUCTION

Managing postoperative outcomes following gender-affirming vaginoplasty (GAV) is crucial to ensuring long-term surgical success and functional outcomes. Although techniques have evolved, loss of vaginal depth and width following full-depth primary vaginoplasty remains a reported outcome across various surgical approaches ( 1 - 3 ). Common tissue options that have been used for restoring neovaginal patency include genital skin grafts or flaps, extragenital skin grafts, intestinal tissue, or peritoneal flaps/grafts. The approach utilized for primary vaginoplasty may inform the tissue options available during revision ( 4 ). Given these limited options, biologically engineered allografts and xenografts are being considered as an alternative to the conventionally used autologous tissue.

Kerecis SurgiClose© (Isafjördur, Iceland) is a decellularized fish skin xenograft (FSX) derived from the North Atlantic Cod skin that has been successfully applied to complex wounds such as diabetic patients, necrotizing fasciitis, and deep skin burns ( 5 - 8 ). This FSX resembles human skin microscopically and has been linked to reduced pain management needs in complex wound care ( 9 ). However, there is a paucity of data on its application as a tissue substitute for revision vaginoplasty in transgender women.

Due to its characteristics, we hypothesized that this graft may be a promising candidate for neovaginal canal lining in revision vaginoplasty. Herein, we report on the surgical technique utilizing decellularized fish skin xenograft revision vaginoplasty (FSXRV) in trans feminine individuals and present clinical outcomes. To our knowledge, this is the largest series describing the use of Kerecis FSX in revision vaginoplasty and the first to demonstrate its use in various graft placement configurations in transgender women.

## MATERIALS AND METHODS

This retrospective chart review included 27 consecutive patients who underwent FSXRV between February 2023 and December 2024. The study was IRB-approved (Protocol #20210504), conducted in accordance with the 2013 Declaration of Helsinki, and all participants provided informed consent.

### Surgical Techniques

Prior to surgery day, a perineal-only versus robotic approach is determined in the outpatient setting based on the degree of width and depth loss. In cases where it was felt that a perineal-only approach would not allow for adequate exposure to create a satisfactorily wide canal all the way to the apex of the neovaginal canal, we opted for a dual perineal-robotic approach. The perineal-only approach typically involves creating a patent introitus with the aid of local skin flaps if necessary. The neovaginal canal is then incised, typically at the 5 and 7 o'clock positions, though this may vary depending on where the exact anatomy of the stenosis. The raw surfaces of the incised areas are then covered with FSX. Depending on the surface area to be covered, the FSX is either cut to size or left intact. The FSX may be sutured to the distal cut edge of the canal and then rotated into the canal with or without additional anchoring sutures. A fully tubularized graft can be inverted into the canal with the aid of a vaginal dilator.

In cases where a concurrent transabdominal robotic approach is utilized, the apex of the neovaginal canal is identified and dissected circumferentially to ensure adequate mobility. The apex is then incised over a dilator. The perineal surgeon works to ensure adequate width of the accessible portion of the canal in the manner described in the prior section. In cases of a particularly short vaginal canal, the FSX may be tubularized and passed to the robotic surgeon. The distal end of the tube is sutured to the cut edge of the neovaginal canal, and the proximal end is sutured to peritoneal flaps raised robotically. As such, this method may require more than one sheet of FSX matrix to line both the incised portions of the vaginal canal as well as extend the canal to ensure adequate depth.

**Figure 1:**
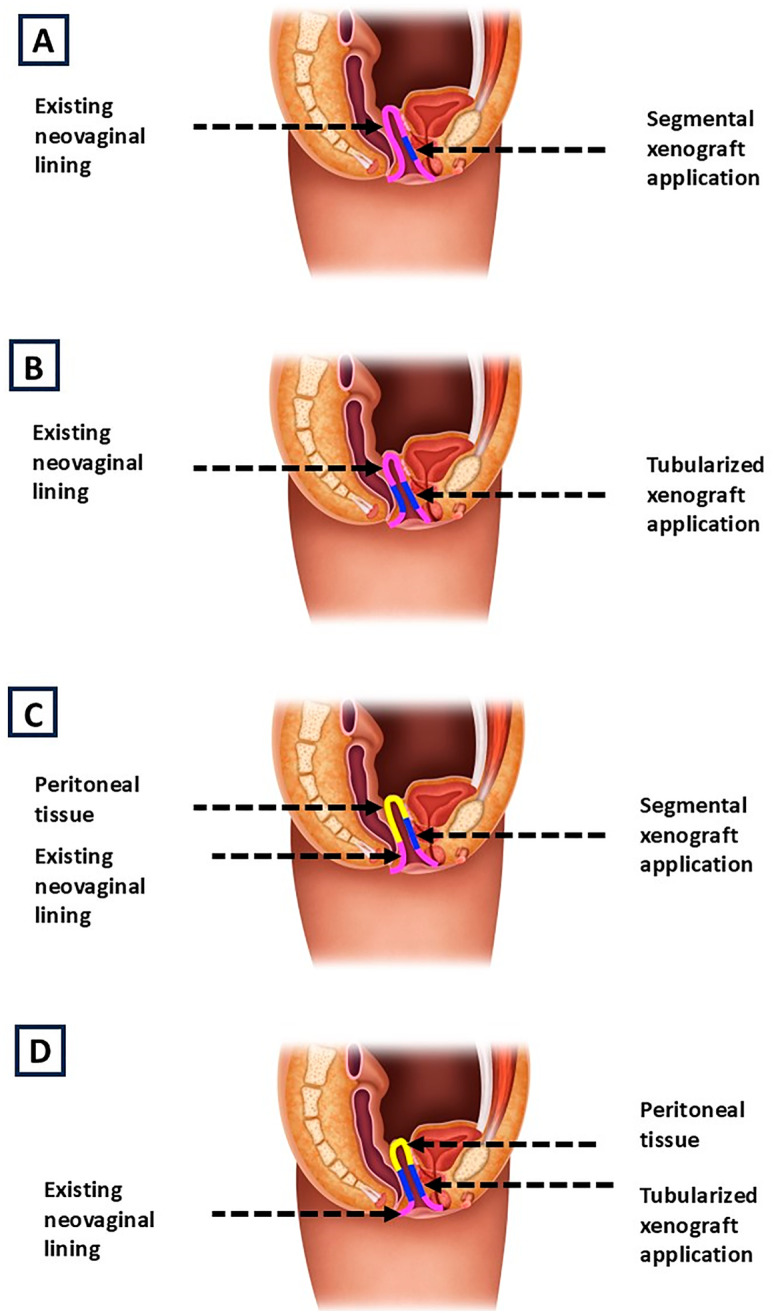
Schematic representation of neovaginal canal reconstruction configurations in revision vaginoplasty using decellularized fish skin xenograft. Pink represents the existing neovaginal canal lining from the index surgery, blue indicates xenograft, and yellow denotes peritoneal tissue. These configurations are not exhaustive and illustrate common combinations used in clinical practice. Segmental xenograft applied along the anterior neovaginal canal wall (A). A tubularized xenograft graft extending from the mid-canal to the introitus, used to augment the existing neovaginal canal (B). Segmental xenograft graft applied to the neovaginal canal combined with robotically constructed peritoneal cap at the time of revision (C). A tubularized xenograft applied from the mid-canal to the introitus, supported by robotically constructed peritoneal cap at the time of revision (D).

**Figure 2:**
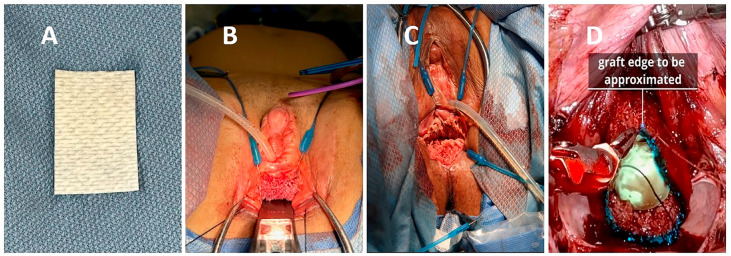
Decellularized fish skin xenograft segment before hydration (A). Decellularized fish skin xenograft used to line the anterior vaginal canal as seen from the perineal view (B). Tubularized xenograft used to circumferentially line the vaginal canal as seen from the perineal view (C) and the intraperitoneal (robotic) view with the proximal edge of the graft highlighted in blue (D). This edge will be sutured circumferentially to peritoneal flaps.

Of note, the FSX matrix is available as a 7×10 cm sheet that expands to approximately 126 cm^2^ when fully stretched. Prior to use, the FSX is soaked in normal saline for a minimum of 30 seconds. As described above, FSX was used in varying capacities based on intraoperative needs. Some patients received a single full sheet or two full sheets, while others had a partial sheet or multiple fragments applied. The grafts were either laid down flat or tubularized ( Figure-1 ). In all cases, a vaginal stent in the form of a rolled wound vac sponge was placed to aid in maximizing surface contact between the graft and the wound bed to encourage graft absorption ( Figure-2 ).

### Data Collection

Data was extracted from patients’ electronic medical records and operative reports. Surgical history included index vaginoplasty type, number and type of interval urogenital procedures, neovaginal canal revision history, and time between index surgery and FSXRV. Revision indications included neovaginal depth loss, width compromise, or graft failure. Intraoperative data captured the surgical approach (perineal-only or robotic-assisted), FSX graft configuration, number of sheets used, graft placement site, pre- and postoperative vaginal depth, depth gain, estimated blood loss, and intraoperative complications.

Postoperative outcomes included hospital stay duration, catheter time, 30-day complications (>Clavien-Dindo II), and 90-day ED visits, genitourinary infections, and readmissions. Subsequent urogenital procedures were recorded and categorized as canal or non-canal revisions, along with time to first canal revision. Patient-reported outcomes included dilation adherence from clinical notes and final follow-up depth based on maximum dilator insertion. Overall patient satisfaction and perceived improvement were assessed through telephone interviews using the Patient Global Impression of Improvement (PGI-I) scale, a validated single-item measure employed to capture subjective treatment effectiveness in gynecological procedures ( 10 ). All data were independently reviewed and verified by two authors to ensure accuracy and completeness.

### Data Analysis

Descriptive statistics were employed to summarize all study variables. Continuous variables were reported as medians with corresponding ranges, while categorical variables were presented as frequencies and percentages.

## RESULTS

### Demographics and Preoperative Characteristics

Patients had a mean age of 40.4 ± 13.3 years (range: 24-68), and a mean BMI of 28.2 ± 5.9 kg/m2 kg/m2 (range: 18.2-39.7) at the time of surgery. Demographics and baseline patient characteristics are summarized in Table-1 . The patients had undergone different types of primary GAV prior to FSXRV. The most common index (primary) technique was perineal-only penile inversion vaginoplasty in 13 patients (48.1%), followed by robotic-assisted vaginoplasty without xenograft (canal lined with peritoneum and penile skin only) in 11 patients (40.7%) and robotic-assisted vaginoplasty with Kerecis FSX (canal lined with peritoneum, fish skin xenograft, and penile skin) in 3 patients (11.1%).

**Table 1:** Demographics and preoperative characteristics of trans feminine individuals undergoing fish skin xenograft revision vaginoplasty.

Demographics and Preoperative Characteristics			
N			27
Age (Mean ± SD) [Range]			40.4 ± 13.3 years [24-68]
**Race, n (%)**			
	White		22 (81.5)
	African American		4 (14.8)
	Asian		1 (3.7)
BMI (Mean ± SD) [Range]			28.2 ± 5.9 kg/m^2^ [18.2-39.7]
**Diabetes Status, n (%)**			
	None		23 (85.2)
	Type I DM		1 (3.7)
	Type II DM		3 (11.1)
**Smoking Status, n (%)**			
	Current		2 (7.4)
	Former		3 (11.1)
	Never		22 (81.5)
History of Abdominopelvic Radiation, n (%)			0 (0)
**Type of Index Vaginoplasty, n (%)**			
	Perineal-only PIV		13 (48.1)
	Robotic-vaginoplasty without xenograft		11 (40.7)
	Robotic-assisted vaginoplasty with xenograft		3 (11.1)
**Interval Procedure? n (%)**			
	No		15 (55.6)
	1		7 (25.9)
	2		3 (11.1)
	>2		2 (7.4)
**Requiring Interval Neovaginal Canal Revision? n (%)**			
	Yes		9 (33.3)
	No		18 (66.7)
**Type of Interval Procedure, n (%)**			Instances [N=20]
	**Neovaginal Canal Revisions**		12 (60)
		Canal revision w/ Integra xenograft only a	6 (50)
		Canal revision with FTSG	4 (33)
		Canal revision w/ Integra xenograft and BMG	2 (16.7)
	**Other Procedures**		8 (40)
		External genitalia/urethral revision	4 (50)
		Introital revision with buccal mucosa	1 (12.5)
		Introital revision w/o graft	1 (12.5)
		Introital revision w/ Myriad xenograft	1 (12.5)
		Fulguration of neovaginal hypergranulation tissue	1 (12.5)
Time from index vaginoplasty to FSXRV (Mean ± SD) [Range]			611.7 ± 561 days [1-2040]

a Integra^®^ graft (Princeton, NJ, USA);

BMI = Body Mass Index; DM = Diabetes Mellitus; PIV = Penile Inversion Vaginoplasty; FTSG = Full-thickness Skin Graft; BMG = Buccal Mucosa Graft; FSXRV = Fish Skin Xenograft Revision Vaginoplasty.

Interventions occurring between primary vaginoplasty and FSXRV were recorded and classified as either canal revisions or non-canal revision procedures. Twelve patients (44.4%) underwent at least one interval procedure. Nine patients (33.3%) required a total of 12 neovaginal canal revisions between primary GAV and FSXRV. The mean time from primary GAV to FSXRV was 611.7 ± 561 days (range: 1–2040 days) ( Table-1 ).

### Operative Characteristics

The most common indication for FSXRV was loss of vaginal depth in 22 patients (79.2%), followed by loss of width proximal to the introitus in 4 patients (14.8%) and devitalized graft in 1 patient (3.7%). The median preoperative vaginal depth was 4 cm (range: 0–11.5 cm).

FSXRV was performed via a perineal-only approach or a combined robotic-perineal approach. The perineal approach was performed in 15 patients (55.5%) and a combined robotic-perineal approach in 12 patients (45.5%). The median postoperative depth achieved intraoperatively was 14.5 cm (range: 9.5–18), with a median depth gain of 10 cm (range: 0–16), regardless of surgical approach. The median depth gain in the operating room using the robotic approach was 10.75 cm compared to 9 cm with the perineal approach. No intraoperative complication occurred in any of the procedures.

### Postoperative Characteristics

The median follow-up duration was 261 days (range: 131–680). No 30-day postoperative complications exceeded Clavien-Dindo grade II. The 90-day ED visit and readmission rates were each 3.7%: one patient (3.7%) presented to the ED for vaginal pain without readmission. One patient (3.7%) was readmitted on postoperative day 70 for an elective canal revision with FSX and labial revision.

Nineteen patients (70.4%) required no further urogenital surgical procedures (canal or non-canal related) following FSXRV. Five patients (20.8%) underwent a total of 7 subsequent neovaginal canal revisions following FSXRV (2 patients each required 2 canal revisions, followed by 3 patients each requiring a single revision vaginoplasty after FSXRV). Revision vaginoplasty with Myriad xenograft (Aroa BioSurgery^®^, Auckland, NZ) was the most common, performed in 3 cases. This was followed by revision vaginoplasty with Kerecis FSX in 2 cases, Revision vaginoplasty with internal pudendal artery perforator (IPAP) flap in one case, and revision vaginoplasty with full-thickness skin graft and IPAP flap in one case. The median time from FSXRV to the first subsequent canal revision procedure was 246 days (range: 70–540 days).

At the most recent follow-up, 25 patients (92.6%) were carrying out regular twice daily dilations. Two patients (7.4%) independently discontinued dilation. One of these patients halted all gender-affirming care following a severe depressive episode. The other discontinued dilation after undergoing vocal cord surgery. Both patients who discontinued dilation had undergone perineal FSXRV. The recorded median vaginal depth at the most recent follow-up was 11.7 cm (range: 0-18).

At the conclusion of follow-up, the median neovaginal depth was 12.3 cm in the robotic group and 11 cm in the perineal group. All 12 patients in the robotic group (100%) maintained regular dilation. In the perineal group, 13 out of 15 patients (86.7%) continued dilation at follow-up. Neovaginal canal revision was performed in 1 of 12 patients (8.3%) in the robotic group and in 4 of 15 patients (26.7%) in the perineal group. Patient Global Impression of Improvement (PGI-I) scores were obtained via telephone interviews conducted at the conclusion of follow-up. Of the 27 patients, 20 (74%) reported positive changes: 8 (29.6%) rated their condition as ‘Very Much Better’ and 12 (44.4%) as ‘Much Better.’ One patient (3.7%) reported ‘No Change,’ and 6 patients (22.2%) did not respond ( Table-2 ).

**Table 2:** Operative characteristics and outcomes of trans feminine individuals undergoing fish skin xenograft revision vaginoplasty.

Operative Characteristics			
N			27
**Indication for Revision, n (%)**			
	Loss of Depth		22 (81.5)
	Loss of Width – Proximal to Introitus		4 (14.8)
	Devitalized scrotal skin graft		1 (3.7)
Preoperative Vaginal Depth (Median) [Range]			4 cm [0-11.5]
ASA Score (Median) [Range]			2 [2-3]
**Type of FSXRV, n (%)**			
	Perineal-Only Approach		15 (55.5)
	Robotic-Perineal Approach		12 (44.5)
**Number of Graft Sheets Used, n (%)**			
	Single sheet		15 (55.6)
	Partial sheet		7 (25.9)
	Unspecified		3 (11.1)
	2 full sheets		2 (7.4)
**Graft Formation, n (%)**			
	One Location		10 (37)
	Tubularized		9 (33.3)
	Multiple Locations		8 (29.6)
**Graft Location, n (%)**			
	Anterior vaginal wall		11 (40.7)
	Circumferentially located between skin and peritoneum		7 (25.9)
	Multiple locations		6 (22.2)
	Vaginal Apex		2 (7.4)
	Posterior vaginal wall		1 (3.7)
Postoperative Vaginal Depth (Median) [Range]			14.5 cm [9.5-18]
Change In Vaginal Depth (Median) [Range]			10 cm [0-16]
Estimated Blood Loss (Median) [Range]			25 cc [5-200]
Intraoperative Complication, n (%)			0 (0)
Follow-up (Median) [Range]			261 days [131-680]
Time with Catheter (Median) [Range]			5 days [0-7]
Length of Hospital Stay (Median) [Range]			5 days [0-7]
30-day Post-op Complication > Clavien-Dindo grade 2, n (%)			0 (0)
90-Day GU Infection, n (%)			0 (0)
90-Day ED Visit, n (%)			1 (3.7)
90-Day Readmission, n (%)			1 (3.7)
**Additional Procedures After FSXRV, n (%)**			
	None		19 (70.4)
	1		5 (18.5)
	>1		3 (11.1)
**Requiring Neovaginal Canal Revision After FSXRV? n (%)**			
	Yes		5 (18.5)
	No		22 (81.5)
**Type of Additional Procedures, n (%)**			[Instances=14]
	**Neovaginal canal revisions**		7 (50)
		Revision vaginoplasty with Myriad^®^	3 (42.8)
		Revision vaginoplasty with Kerecis^®^	2 (28.5)
		Revision vaginoplasty with IPAP	1 (14.2)
		Revision vaginoplasty with IPAP and FTSG	1 (14.2)
	**Other procedures**		7 (50)
		External genitalia/urinary tract revision	5 (71.4)
		Neovaginal hypergranulation tissue excision/fulguration	2 (28.6)
Time from FSXRV to First Subsequent Canal Revision Procedure (Median) [Range]			246 days [70-540]
**Regular Dilation at Follow-up Conclusion? n (%)**			
	Yes		25 (92.6)
	No		2 (7.4)
**PGI-I Satisfaction Survey n (%)**			
	Very much better		8 (29.6)
	Much better		12 (44.4)
	A little better		0 (0)
	No change		1 (3.7)
	A little worse		0 (0)
	Much worse		0 (0)
	Very much worse		0 (0)
	Unspecified		6 (22.2)
Vaginal Depth at Most Recent Follow-up (Median) [Range]			11.7 cm [0-18]

ASA = American Society of Anesthesiologists; GU = Genitourinary; ED = Emergency Department; IPAP = Internal Pudendal Artery Perforator flap; PGI-I = Patient Global Impression of Improvement

## DISCUSSION

Revision vaginoplasty may be warranted for trans feminine patients presenting with complete or partial loss of neovaginal depth. A systematic review of 59 studies with a pooled patient population of 7338 transgender individuals, indicated that the risk of vaginal stenosis following primary GAV across a number of approaches is approximately 5.83%. This rate increases to 9.68% when cases of introital stenosis are accounted for ( 11 ). Surgical correction of a stenosed neovaginal canal often requires repeat access to the rectoprostatic space and lining defects with available tissue options. ( 12 ) Our study aims to address the latter challenge, which is secondary to the limited availability of autologous grafts/flap ( 13 , 14 ).

In cases where genital skin is insufficient for revision GAV, alternative tissue sources have been utilized, though each presents distinct limitations. Options such as extragenital skin grafts may be associated with scarring and inflammatory responses in both donor and implantation site ( 4 ). Studies evaluating revision colovaginoplasty have shown outcomes such as malodor, mucus secretion, and inflammatory colonic complications ( 15 , 16 ). While newer robotic peritoneal revision vaginoplasty techniques have shown promising outcomes, some cases require additional tissue options to augment the neovaginal lining ( 17 ). Allografts and xenografts offer the advantage of avoiding natal tissue harvest and preoperative hair removal; however, their adoption in revision vaginoplasty remains limited ( 18 ).

To our knowledge, the only previously published application of Kerecis FSX in revision gender-affirming vaginoplasty is a single case report involving a 38-year-old transgender woman, assessing short term reliability ( 19 ). Our cohort represents the largest series to date augmenting the natal tissue with decellularized FSX for revision procedures with a significant follow-up period. Another example of successful decellularized graft application in revision vaginoplasty is the use of AlloDerm^®^ (Branchburg, NJ, USA) cadaver-derived matrix. The study included 9 patients with neovaginal stenosis who had undergone robotic peritoneal vaginoplasty augmented with decellularized matrices to bridge the gap between skin and peritoneum. All 9 patients were reported to be successfully dilating at 1-year follow up which was similar to the 100% compliance rate of our robotic FSXRV patients ( 17 ).

Another biological tissue that has been studied in the context of revision GAV is Integra^®^ graft (Princeton, NJ), a bilayer tissue substitute from bovine and shark derivatives ( 20 ). A case series of 9 patients reported the outcomes of a two-step approach with Integra^®^ implantation followed by delayed full thickness skin grafting for revision GAV in patients with canal stenosis and loss of depth. In contrast, the FSXRV method could be done in a single procedure ( 21 ).

We opted to use decellularized FSX in cases where the native genital tissue was insufficient for neovaginal reconstruction. This decision was made after observing evidence suggesting that these xenografts may offer superior elasticity and faster epithelialization compared to conventional full-thickness skin grafts in deep wounds ( 22 ), potentially making them a promising option for neovaginal canal lining. Another contributing factor to this decision was that one-third of our cohort had undergone a prior neovaginal revision between the primary vaginoplasty and FSXRV, reflecting the complex surgical history and limited native tissue available for reconstruction.

The FSXRV technique enabled us to perform revision vaginoplasty on patients without being limited by factors associated with their previous gender-affirming surgical procedures. We were able to demonstrate the feasibility of this approach using both robotic and perineal approaches. This graft was successfully utilized even in patients with prior peritoneal vaginoplasty by constructing a peritoneal cap that was augmented with xenograft. From a surgical standpoint, FSXRV offers the advantage of incorporating xenografts in customized shapes, allowing them to be precisely tailored to the anatomic dimensions of the neovaginal canal. In addition, our findings suggest that decellularized fish skin xenografts are linked to a low rate of early postoperative complications. No major complications necessitating surgical intervention were observed within the first 30 days, and there were no genitourinary infections reported in the first 90 days postoperatively.

Individuals undergoing subsequent revisions after revision vaginoplasty represent the more complex subset of patients as multiple tissue options for neovaginal lining may have been already exhausted. In the case of FSXRV failure, we opted to use Kerecis^®^ matrix, Myriad^®^ matrix (Aroa biosurgery, Auckland, New Zealand) derived from ovine collagen ( 23 ) or apply alternative surgical techniques such as using IPAP flaps for neovaginal revision ( 24 ). In our experience, using decellularized fish skin xenograft does not limit the use of other canal lining options in future revision procedures, and previous use of other grafts does not prevent the use of decellularized fish skin xenograft.

### Limitations

The sample size in this study was limited, which necessitates long-term follow-up and controlled comparative studies with other graft options. Future studies comparing the Kerecis xenograft with other commercially available graft options will help determine the most viable option for revision vaginoplasty in the trans feminine population. The next phase of our study will include evaluating neovaginal sensation, sexual function, determining histological changes on the xenograft at various time points, and comparing the efficacy of Kerecis FSX with other available graft options. Another limiting factor in our study was the 22.2% non-respondent rate to the PGI-I questionnaire. Considering that we were limited to a telephone-only approach during working hours for the PGI-I survey based on the IRB-approved protocol, participants were deemed uncontactable for survey purposes after three unsuccessful attempts within a two-week period following the first telephone contact.

## CONCLUSIONS

Decellularized fish skin xenograft application for neovaginal canal lining is a promising solution for transgender women undergoing primary or revision GAV procedures with a minimal learning curve. The physical properties of the xenograft allow for its application in various locations and formations in the neovaginal canal, facilitating its application in a variety of clinical scenarios. Longer term and comparative studies are required to address the safety and efficacy of this graft in comparison with other conventionally used tissue options.

## Data Availability

The data that support the findings of this study are available upon reasonable request.
